# A Memoir on the Yellow Fever of the West Indies, *as It Occurred in the Year* 1838, *at St. Pierre, Island of Martinique*

**Published:** 1840-01-18

**Authors:** E. Rufz

**Affiliations:** Adjunct Professor at the School of Medicine of Paris


### MEDICAL EXAMINER.

DEVOTED TO MEDICINE, SURGERY, AND THE COLLATERAL SCIENCES.

No. 3.] PHILADELPHIA, SATURDAY, JANUARY 18, 1840. [Vol. III.

### A MEMOIR ON THE YELLOW FEVER OF THE WEST INDIES,

*As it occurred in the year* 1838, *at St. Pierre, island of Martinique.* By E. Rufz, D. M. P., Adjunct Professor at the School of Medicine of Paris.

[Translated for the Medical Examiner, from the French Manuscript.]

The last epidemic of yellow fever in Martinique occurred in the year 1826. Since that time the bills of health, with which ships going to France are obliged to be provided, have been given to them as clean bills, i. e., expressly stating, that the yellow fever did not exist in the island at the time of their departure.*

The disease, however, showed itself sporadically at every season: I have myself seen many cases, while going at different times through the wards of the military hospital, and I have been informed by M. Catel, the physician of the establishment, that it was not an uncommon thing. These sporadic cases were considered to be less grave than the epidemic. In the month of July, 1838, accounts received from Guadaloupe,† brought information that there prevailed in that colony a disease called, by some, *Yellow Fever*,—by others, *Typhoid Affection*,‡—but always occurring under a very

* Thus it appears that the colony was spared for nearly twelve years. The epidemic of 1826 had commenced in 1816, and continued during ten years, with different degrees of severity. The years 1816 and '17, according to M. Dariste, (Recherches sur la Fiévre Jaune,) and 1821, '22, and '23, according to the information I have obtained from resident physicians, were those in which the disease made the greatest ravages.

From 1816 to 1806 there was an intermission: but between 1806 and 1802 there existed a very fatal epidemic. In going back, we find another intermission from 1802 to 1797, and from 1797 to 1794 another epidemic. An account of the order of succession of these different epidemics may be seen in the work of M. Dariste, who resided in the colony from 1791 to 1819, a period of nearly thirty years, pages 19, &c.

The occurrence of intervals between the epidemics of yellow fever is a remarkable fact, which did not even escape historians who were unconnected with medicine: it was noticed by Father Labat. If we search the history of the times, in order to ascertain what circumstances may have given rise to the appearance of the yellow fever at these epochs, we find no remarkable peculiarity, except the presence of a great number of unacclimated persons. Thus, the epidemic of 1794 coincides with the conquest of the island by the British; that of 1802 with the surrender of the island to the French, by the treaty of Amiens; that of 1816 with the surrender of the island to the French, by the treaty of Paris. All these captures and re-captures required movements of great masses of unacclimated men who had come from Europe.

In the epidemic of 1838 we see nothing similar, unless we choose to consider as such our war with Mexico, which may have tended to excite the fever on the shores of the gulf, at Vera Cruz, which is, as it were, its capital. But the French expedition had no communication with the Windward Islands.

† This time the epidemic passed through Gaudaloupe before it reached Martinique: it was the same case in 1816, (see Dariste, page 23.) Gaudaloupe is in latitude 16° North, Martinique 14°. I have been unable to decide whether this course of things has always taken place or not: whether the other epidemics in every case commenced at Gaudaloupe, that is to say, whether the yellow fever always proceeds from North to South, or from West to East, in the same way as the cholera advanced from East to West. I recommend this fact to the attention of the Academy, and especially of Dr. Chervin, the zealous historian of yellow fever. By the documents which I have been able to procure here for the study of this point, I see that the disease, before it reached us in 1838, had made its appearance in 1837 at New Orleans, after a truce of four years, (Rapport de M. Chervin sur trois Lettres de M. Thomas:) in 1836 it existed at Tampico, which is in latitude 22°, (Rapport de M. Chervin sur un Mêmoire de M. Grupilleau:) in 1837 it was epidemic at Havana, (Rapport de M. le Docteur Maker au Ministre de la Marine, Archives Maritimes.) All these places are to the north and west of Martinique. On the contrary, in the islands to the south, St. Lucia and Barbadoes for example, the epidemic did not show itself until after its appearance in Martinique, of which I am assured by letters from Drs. Clavier, of St. Lucia, and Evans, of Barbadoes.

But at Trinidad, Dr. De Verteuil writes me, that an epidemic, quite similar to that of Martinique, existed in May and June, 1838, *i. e.* nearly at the same time that it appeared in Guadaloupe. Trinidad is in latitude 10°. According to Dr. Tholozan, (Thèse de Montpellier, No. 20, 1838,) one would have thought that the disease had not existed in Trinidad prior to the year 1817. At this time there was an epidemic in that island. It is now quite certain that the epidemic has actually prevailed there.

Cayenne has the character of being exempt from the yellow fever. The same is probably true of Demarara.

‡ The names which have been applied to yellow fever, and, indeed, to all the diseases ranged under grave form, and attended by a great mortality. The accounts from that island becoming more and more unfavourable, the board of health of the city of St. Pierre decided by a resolution of the 15th of July, that ships from Guadaloupe and Demarara* should proceed to Fort Royal, and there undergo a quarantine.†

In spite of these precautions, on the 16th of September, M. Pouvreau, surgeon of the regiment, recognised a case of yellow fever in the garrison. From this time the cases multiplied; but it was on the 6th of October that the epidemic firmly established itself. Before this epoch, from the 1st to the 6th, but nine patients had entered the hospital. From the 6th to the 15th the number of deaths amounted to twenty-four. In the whole month of October, only sixty-eight were received. There was then, without any doubt, an epidemic prevailing. The patients all offered nearly the same symptoms: face red, and almost violet colour, (this colour disappeared under the pressure of the finger and was slowly re-established.) The conjunctiva was injected; the pupils very often dilated; cephalalgia and weight in the anterior part of the head, almost immediately above the eyebrows: tongue coated white, except at the edges where it was red and moist; no thirst, some nausea, and in a small number, vomiting; abdomen soft, and without pain; skin coloured, warm and moist; pulse full, soft and regular, from one hundred to one hundred and eight.

These symptoms began suddenly, but were preceded in a majority of cases by a chill, sometimes by a state of *malaise* and of anorexia, which lasted two or three days.

In the greatest number of patients, these symptoms disappeared after repeated bleedings; several had hæmorrhage from the nose, mouth, or bowels.

Up to the 15th of October, only four patients

---

the head of *Fevers*, have varied, at different times, according to the manner in which they have been considered. It is thus that yellow fever has been designated as *putrid fever*, *malignant fever*, *gastro-enteritis*, &c. The word Typhoid being the last term applied to the fevers of Europe by modern nosologists, who have written since 1827 or '28; and an opportunity of applying the new term not having presented itself, as the yellow fever had not existed in the colonies since that epoch, is it surprising that on the return of the disease at the present time, attempts have been made to apply the fashionable term? As if a new word was a new source of knowledge. Is it not in this way that the human mind often proceeds? This identification of the yellow fever of America with the typhoid fever of Europe, although adopted by the physicians of the country, is not correct, and may lead to error. The details which we shall enter into, willshow that the epidemic which has prevailed in Martinique, offers no point of resemblance to the typhoid fever of Europe, as regards either its symptoms, or the anatomical lesions attending it. The differences between these two diseases are so well marked, that I consider it useless to point them out.

* Since the beginning of 1838, our intercourse with Demarara had become very great, in consequence of an emigration of our labouring class, who chose to quit Martinique, rather than subject themselves to taking out a passport from the police. These unfortunate persons had not found at Demarara, the object of their fond hopes, fortune without labour, and they returned in great numbers, a prey to every species of misery. It was for this reason that vessels returning from Demarara, were also subjected to quarantine. I saw many of these emigrants with intermittents, but not one had had the yellow fever. It is not useless to explain such facts as these, which may serve to combat the doctrine of acclimation, and to sustain that of contagion, two errors at the present day untenable.

Already M. Dariste had thought that he ought to correct, in his work, a mistake with regard to a fact quite similar, which had been in his time misinterpreted by some writers, (page 18.)

"They had stated that the inhabitants of Martinique and Guadaloupe, who had emigrated in 1793 and 1794 to the colonies of Dominique, St. Vincent and Grenada, had been attacked by yellow fever, and that eight hundred of them, as well as many of their negroes, had fallen victims to it. Nothing," adds M. Dariste, "can be less correct than these assertions; the yellow fever attacked neither the colonists nor the acclimated. I saw, indeed, prevailing among them, remittent and intermittent fevers, which were also aggravated by the depression arising from their misfortunes, and moreover by an epidemic of measles, which, more particularly, attacked the negroes, whom they were compelled to feed on half putrid salt fish: but no one, absolutely no one, was seized with the yellow fever; and this is the account which history gives."

Will they not in some years from this time write, that the yellow fever has been brought to us by the return of the labourers, who had emigrated to Demarara? This is now the popular opinion, and it might be supported by the resolution of the board of health of the Island, which subjects ships from Demarara to a quarantine. And I repeat, with M. Dariste, that absolutely not one of them was seized with yellow fever.

† After so many proofs and corroborations of the non-contagion of yellow fever, can any one conceive that the government will persist in maintaining quarantines? What further knowledge is necessary to enlighten them on this point? They have given up quarantines for cholera; and at Havre, Bordeaux, and Marseilles, they lengthen, by eight or ten days, the long and real quarantine of from thirty-five to forty days, which are requisite to cross the ocean. And for the height of folly it suffices, that a ship shall touch at a port in England for twenty-four hours, and be exempt from this vexatious measure. She is then considered as coming from England. Some of our merchant captains do this when they are carried by the winds to the coast of England. They touch there to avoid a quarantine at Havre.

died at the hospital,—these had become yellow before their death. This was at the military hospital. In the city, we had severe fevers from the month of September.

On the 20th of September, M. Virginy, clerk of the court, creole, aged thirty years, died of a continued fever, which, in the last hours of life, assumed the characters of malignant remittent. He did not become yellow after death. He was visited by one of my brethren, M. Noverre.

About the same time I had three cases of congestive fever, which I will detail here.

OBSERVATION I.

Mademoiselle Zamée Closet, æt. fifteen, fat and lymphatic form of body, born in the country, but for nearly a year placed at the convent of the ladies of St. Joseph; irregular menstruation, but in other respects in good health.

On the 13th of September she felt indisposed, (the menses ought to have appeared five or six days before,) with fever and intense headach, but no other disturbance of her functions.

On the evening of the 15th I was called. I found her in the following condition:—Face a little flushed; cephalalgia rather intense; tongue red at the point, but in the rest of its surface thickly coated; little thirst; no nausea or vomiting from the beginning; abdomen soft, and not tender; neither diarrhœa nor constipation; no cough nor sore throat; moderate heat of skin; no eruption; pulse one hundred and twenty.

Her condition remained the same to the 19th, characterized only by the frequency of the pulse, and the headach, which is somewhat variable as to its intensity. But, although I visited the patient both morning and evening, I could not detect any real remission. I inquired of her at every visit, and learned that she had not experienced any chill; it is true, she was extremely timid, and gave a bad account of her symptoms. Not being able to refer this succession of symptoms to any local lesion, I concluded that the amenorrhœa had some influence upon them, and with this view I directed the treatment. On the 17th thirty leeches were applied to the groins, with pediluvia, hot enemata, fumigations to the vulva, and emollient tisanes.

The leeches bled freely, and their application was followed by fainting. The 19th, same frequency of the pulse; the patient complains of some palpitation. In other respects she was so well, that they thought of sending her to her parents, who wished her to return to them in the country, seven leagues from town. This journey was prevented by bad weather; but thinking that she had gone, I did not again visit her.

On the evening of the 20th, I was informed that, after having been pretty well during the day, she had had a return of fever, with more heat of skin and headach. During the night there was a little delirium.

*21st.*—Less injection of the face; cephalalgia much less; strength pretty well returned, and intelligence perfectly clear. The patient is able to sit up. Less redness of tongue, and thirst. Some tenderness of the epigastrium, with an uncomfortable feeling complained of by the patient. The skin is moderately warm, but the pulse is tense, frequent, one hundred and twenty-four. This state of the pulse, which does not accord with the general calmness, is the only dangerous symptom. (Twenty leeches to the groins; in other respects, treatment as before.) The day was calm,—the leech-bites bled freely. Towards 3 o'clock, the patient began to feel a coldness of the extremities, which increased every moment, and was attributed to the flow of blood, which still continued. I was informed of it at 7 o'clock. I found the patient with cold extremities, but the face warm, and the whole body covered with a clammy sweat. The cephalalgia continued; the intelligence was clear; strength preserved; expression calm; tongue moist, and almost natural; no thirst; abdomen depressed, yielding; no urine; pulse almost insensible, intermittent.

The leech-bites bled until they were cauterized. Twenty-four grains of quinine were directed to be given during the twenty-four hours, with an enema containing forty-eight grains, with twenty drops of laudanum. Two blisters to the thighs; flying sinapisms to the lower extremities.

Although the patient retained the potion and the enema, she did not react, but, on the contrary, the chill increased. The pulse was altogether insensible at 10 o'clock; however, the strength was retained, and the countenance was calm. Without touching the patient, the gravity of the mischief would not have been suspected. At midnight she ceased to answer questions; at 3 o'clock in the morning, there were some convulsive motions, with cries resembling an hysterical attack. Death occurred at 3½ A. M.

Twelve hours after death the body was cold, but neither bluish nor yellow. No autopsy was made.

I believe that this disease was certainly a congestive intermittent fever, with no appreciable intermission, unless the amelioration occurring from the 19th to the 20th should be regarded in this light. But, every time that I saw the young girl, her pulse was frequent, and I was obliged to give the sulphate of quinine during the febrile state, from the impossibility of finding a propitious time.

No other cases of the same kind occurred in the convent; the rains during the course of this month had been very abundant.

CASE II.

M. Roussel, æt. nineteen, of a moderately strong constitution, and a melancholy temper, had been three years in the colony. He had had a long continued dysentery,—and, from that time, his health was not as good as before. He was fond of taking physic, which was an easy thing for him, as he was an apothecary's pupil.

Towards the 15th of September, he felt during three or four days, much malaise; lost his appetite; on the 17th, diarrhœa, with colic. On the 19th he took an emetic, with ipecacuanha, which produced seven or eight stools, and arrested the diarrhœa. During these days he had felt each evening some agitation, and more or less febrile heat. On the 21st, in the evening, there was an intense chill, followed by high fever and abundant sweat. I saw him on the morning of the 22d.

Eyes hollow and injected, a little haggard; cephalalgia intense; tongue whitish, and coated, without any redness; little thirst, complete anorexia; no pain in the epigastrium; neither nausea nor vomiting; abdomen yielding; no diarrhœa; no urine passed since yesterday evening; no cough; sweat abundant over the whole body; skin moderately warm; pulse one hundred and twenty, and corded.

I thought I saw in M. Roussel's case an affection similar to that of Mlle. Zamée Closet, viz., a malignant remittent fever at an advanced stage. Consequently, I prescribed a solution of gum, with twenty-four grains of sulphate of quinine, to be taken in twenty-four hours.

He bore this prescription well. The sweats continued to be very abundant during the 22d, 23d, and 24th. The cephalalgia, on the contrary, diminished, and the pulse lost its frequency. On the 23d, during the day, it was eighty-four.

A new solution, with thirty-eight grains of sulphate of quinine, was ordered on the 24th. In the evening there was a slight acceleration of the pulse, which rose to ninety-two. There was no cephalalgia, and the skin was a little dry.

25*th.*—Continual vigilance; tenderness in the epigastrium; saliva thick, and difficult to swallow; some nausea and vomiting after taking a custard; constipation followed by six or seven stools, (after an injection of five spoonsful of olive oil;) abdomen yielding and natural; skin moist; pulse eighty-four, and regular. This state continued for some days following; there was joined with it likewise a sufficiently apparent stupor; the eyes were injected and haggard, and the countenance was expressive of suffering. The patient, moreover, complained of pain in the epigastrium. The pulse, which, in the morning, was eighty-four, arose towards evening to ninety-six, and subsequently to one hundred and six; it was all the time very much corded.

I dared not again have recourse to the sulphate of quinine. On the 27th I put twenty leeches behind his ears. The patient continued to take emollient drinks, and also broths and custards.

On the 29th, the state of stupor not being changed, I ordered two blisters to the legs. From this time the stupor diminished. The patient got better without any appreciable critical affection. The tenderness in the epigastrium only continued, and he had frequently symptoms of nausea.

His convalescence was protracted and very painful, although he was sent into the country for a change of air. As I had learned that he had some moral disturbance, I directed him to go to France, his native country. While waiting for the time of his departure, he continually dosed himself with purgatives. He had no appetite, grew thin, and his skin assumed a yellow tinge; he had fallen into the most gloomy hypochondriasis. The 7th of December he obtained his passport, in order to set off the next day, ate some soup towards evening, and appeared cheerful enough. During the night he was heard to complain; at 7 in the morning he was seized with a very copious vomiting of black blood, and almost at the same time he also discharged a large quantity of the same kind of blood per anum. His conjunctivæ and the whole of his skin became yellow; his eyes assumed a haggard appearance; he got up frequently, and appeared to be in a state of delirium; his pulse was thread-like. The vomitings and stools of blood were repeated in the day; subsultus tendinum and frequent faintness took place. At noon he had the tracheal rattle, and he died at 7 in the evening. He was not examined.

Thus, in September, that is to say, at the commencement of the epidemic, Roussel had a malignant remittent fever,—and in December, one of the periods in which the epidemic manifested the greatest severity, he succumbed to a violent attack of yellow fever; for I think that no one can consider in any other light the series of symptoms experienced by him towards the last. I was then surrounded with similar cases.

This case deserves some attention, inasmuch as it shows an individual, acclimated for three years, enfeebled by a long continued dysentery, and more recently by a malignant fever, nevertheless contracting the yellow fever. This disease, then, does not depend solely upon the richness of the blood of Europeans, as it is said to do; it is an influence to which it is necessary to be acclimated.

CASE III.

Mademoiselle Alexandrine Giron, aged 25, of a strong constitution, had resided in the colony for a year, had always enjoyed good health. On the 15th of September she was seized with great uneasiness and pain in the head, with loss of appetite. These symptoms continued three days; on the 17th she took an emetic, and then, not having obtained any relief, she was bled; the day after the bleeding she took a purgative of manna, and the day after, which was the 19th, a purgative, with a quack's powder, called *poudre de Labouree.* During the treatment, the symptoms, so far from being ameliorated, were aggravated. On the 24th, two blisters were applied to her legs.

On the morning of the 28th, she called me in. The cephalalgia had continued the whole time; her eyes were half shut; her face was of a violet colour, which disappeared on pressure, and afterwards returned very slowly. She also had a very marked expression of stupor; her answers were slow; her hearing dull; decubitus dorsal; tongue somewhat smooth, without redness or dryness, pale and clear; there was no thirst. The patient had not had either nausea or vomiting; the abdomen was yielding and free from pain; bowels rather relaxed than constipated; there were two or three stools a day. The limbs were cool, and often covered with a lukewarm sweat; the pulse small, compressible, and regular. The persons who were about the patient informed me, that there were, during the day, very marked exacerbations of the symptoms; that the skin at the time became more red, the sweat more abundant, that the faintings were more frequent, and that there was often conjoined, with all these, a little delirium.

I prescribed twenty grains of sulphate of quinine, to be taken in the course of twenty-four hours, at intervals of two hours, and in conjunction with this, an enema of cinchona.

The symptoms were the same during the days from the 28th to 30th. I only remarked that the abdomen was a little meteorised after the enema, and that the exacerbations were less frequent, and not so violent.

On the 30th there were some twitchings of the tendons. I suppressed the cinchona.

From the 1st to the 4th of October, the state of the patient was somewhat ameliorated; the countenance was a little more open; the subsultus diminished, and finally disappeared; the pulse was 112. The exacerbations became less appreciable, but the faintings were still frequent; the skin was always moist and sticky; the urine was sometimes discharged involuntarily, but was never suppressed; some sudamina existed on the neck.

On the 4th there was a remarkable amelioration; the patient took more notice of what was passing around her, the countenance had less of the violet colour; there were still some faintings; the tongue had always remained white and moist; the abdomen was yielding, and the bowels free. A miliary eruption appeared upon the arms and back.

From the 30th, the patient had taken nothing but barley water.

From the 4th, there was an amelioration from day to day.

On the 7th, she had severe pains about the lower jaw, which continued during the day; these pains were removed by the use of emollient poultices.

On the 10th the convalescence was complete; on going into the country for change of air, she fell into a state of mental alienation, which lasted near a month, but was at last entirely removed.

This case is not, like the two preceding, one of malignant remittent, nor is it one of yellow fever, as we shall presently see. This variety would justify the term typhoid, if we trust this as designating merely a particular combination of symptoms.*

It was in this manner that the epidemic seemed to be prevailing in the town. These cases, although grave, were very different from those at the hospitals, no patient died in a chill, as Mademoiselle Closet, and no one had such profuse sweats as M. Roussel. Mademoiselle Alexandrine Giron was not seen by me until a late period of the disease. She offered, in the progress of the symptoms, a condition which I have since seen in a young European, Mademoiselle Eliza, (see case.) This state seemed to me to be one form of yellow fever.

In these three cases it is unquestionable that the sulphate of quinine was of service.

We must, however, remember that the city of St. Pierre has no endemic intermittent fevers. Situated directly on the sea, at the foot of high hills, it is freely ventilated by every wind. There are no marshes in the adjacent country. The sea, which washes the city, forms an open roadstead, and has no creeks of stagnant water. The bottom is every where sandy, and the water is kept perfectly clear by the rising and falling of the tide. The streets of the town are broad and well paved. The gutters are kept perfectly clean by streams of water, which are conducted through them towards the sea. This arrangement is the more easy from the plan of the city, which is built like an amphitheatre. The houses are large, well-aired, and more numerous than is required for the population.

However, towards the second half of the winter season, after the rains, we found that several diseases which, at their origin, were

* I have not reported these cases as examples of yellow fever, but as facts which may have some bearing upon the etiology of the disease. Was the same morbid influence at work upon the two classes of the population, taking on an intermittent form in individuals who were acclimated, and a continued one in those who were not? How far can this comparison of these diseases render their nature and treatment more intelligible?

When all my facts have been related, I will attempt to answer some of these questions.

continued, became intermittent, and required the use of sulphate of quinine. During the same time that I noted the cases of Mademoiselle Closet and M. Roussel, I met with three or four of these affections.

As to fevers becoming really congestive, after three or four paroxysms, I have never seen a case occur at St. Pierre before that of Mademoiselle Closet. Every case of the kind occurred in individuals who had left other parts of the island, where those fevers are endemics, within fifteen or twenty days previously to their attack.

The last epidemic which we had had at St. Pierre, was whooping-cough; it was slight, and lasted from September, 1837, to March, 1838. Since that time we have had the diseases of the seasons. In June and July, rheumatic pains, furuncles; in August and September, gastric *embarras*, and some badly marked ephemeral fevers.

I have already stated that the yellow fever began in the garrison towards the end of September. After the three severe fevers which I have already mentioned; the first case which I observed in the city, in which the nature of the disease was very evident, was that of M. Beligny, on the 10th of October. He was a young man of robust constitution, intemperate, a native of Martinique, but he had passed twenty years in France, from which country he had only returned a year before. He lived about five or six minutes walk from the city hospital, where the sick soldiers were, but he had had no communication with that institution. He recovered.

The second case was that of M. Fraquet, on the 27th of October. He lodged at the other end of the town, near the fort, far from the barracks, the harbour, and the hospital; that is, from every place which could be regarded as an infected point. He was a teacher in a boarding school, led a retired life, and had no communication with the town.

It is, then, worthy of notice, that the disease began simultaneously at the anchorage and at the fort, two quarters of the town which are placed in very different hygienic circumstances. The anchorage is protected by high ground, and the fort is at the entrance of a valley, where there are continual currents of air.

I will now detail the case of M. Fraquet.

[*To be continued.*]

